# P-2016. Optimizing Statin Use for Primary Prevention of Atherosclerotic Cardiovascular Disease in People Living with HIV

**DOI:** 10.1093/ofid/ofaf695.2180

**Published:** 2026-01-11

**Authors:** Cherry Maung Maung Aye, Usha Rathi, Parversh Kumar Rathi, Khine Nyein Chan, Myat Wint Thu, Tina Zheng, Samantha H Cham, Yan Naing Tun, May Thet Hmu Tun, Yu Shia Lin, Edward Chapnick, Monica Ghitan

**Affiliations:** Maimonides Medical Center, Brooklyn, NY; Maimonides Medical Center, Brooklyn, NY; Maimonides Medical Center, Brooklyn, NY; Maimonides Hospital, New York, New York; Maimonides Medical Center, Brooklyn, NY; Maimonides Health, Brooklyn, New York; Maimonides Medical Center, Brooklyn, NY; Maimonides Medical Center, Brooklyn, NY; Maimonides Health, Brooklyn, New York; Maimonides Medical Center, Brooklyn, NY; Maimonides Medical Center, Brooklyn, NY; Maimonides Medical Center, Brooklyn, NY

## Abstract

**Background:**

Cardiovascular disease (CVD) - related deaths have doubled in people living with HIV (PLWH) as antiretroviral therapy extended their life expectancy. Atherosclerotic cardiovascular disease (ASCVD) risk calculators may underestimate CVD risk in PLWH. The REPRIEVE trial recently confirmed that statin use lowers the incidence of major adverse CVD events in PLWH. This leads to the US Department of Health and Human Services (HHS) updated guidelines recommending statin therapy for the primary prevention of ASCVD in PLWH.

**Table 1:**
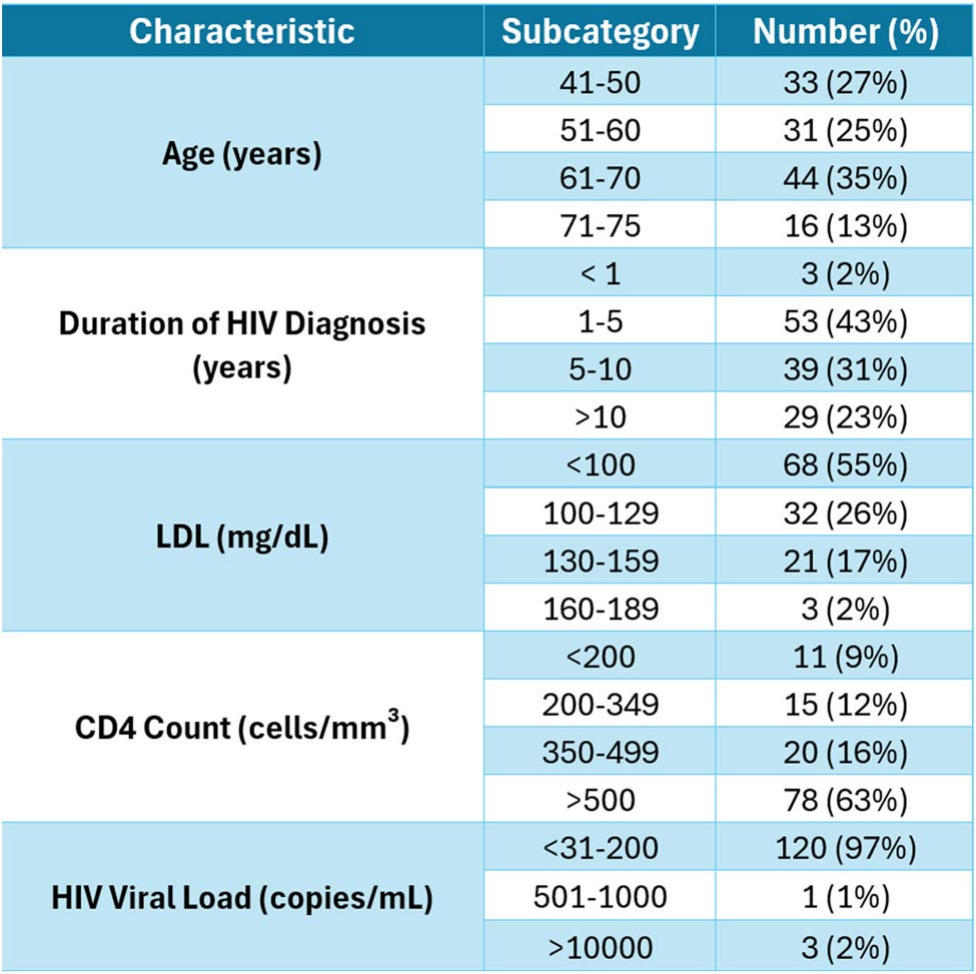
Patient Characteristics

**Figure 1:**
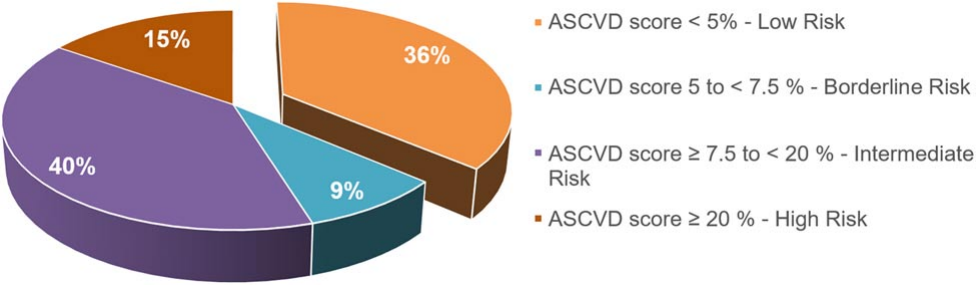
Percentage of Patients by ASCVD Risk Score

**Methods:**

We conducted a retrospective chart review of PLWH aged 40–75 who received care at our HIV practice between January 1, 2022, and June 30, 2024. Eligible patients were on active antiretroviral therapy and had at least two follow-up visits, an available lipid panel, and no history of ASCVD. This study aimed to determine the proportion of patients receiving appropriate statins for primary ASCVD prevention and identify reasons for statin underuse.

**Figure 2:**
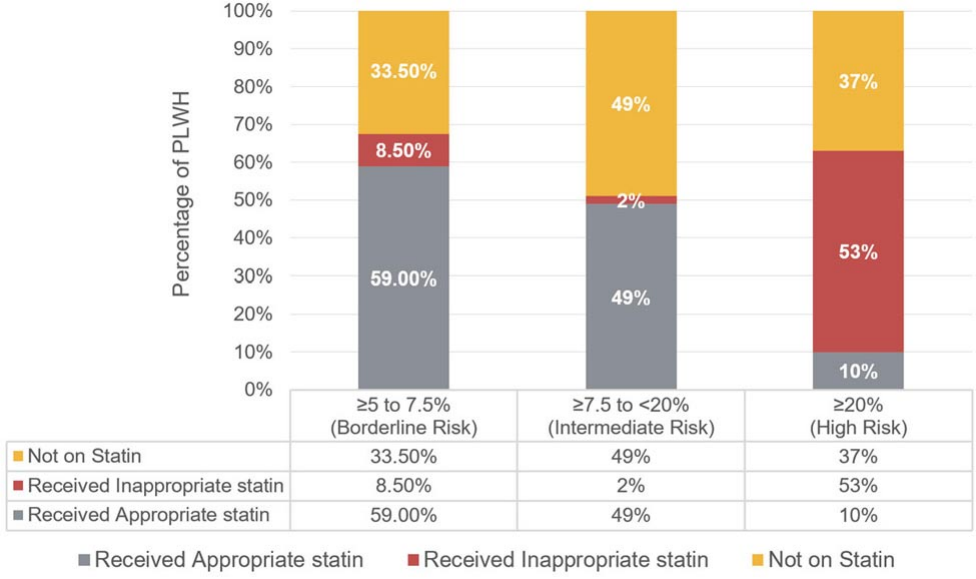
Percentage of Patients Not Receiving Statins, and Receiving Inappropriate and Appropriate Statins Across Different ASCVD Risk Groups

**Results:**

A total of 124 patients aged 40-75 years were included, with 57% male. The ethnic breakdown was 38% African American, 31% Caucasian, and 17% Asian. Comorbidities included hypertension (52%), diabetes (21%), and smoking (21%). Table 1 presents patient characteristics, while Figure 1 illustrates the distribution of ASCVD risk scores among them. Figure 2 illustrates the percentage of patients who received appropriate statins across different ASCVD risk groups. Of 124 patients, 80 (64%) had an ASCVD risk score ≥ 5%, for whom statin therapy was indicated. Only 45 of these patients (56%) were prescribed statins, and 27% of those did not receive the appropriate intensity. 44% of those above 80 PLWH were not prescribed statins, with physician non-prescription accounting for 95% of these cases. Among the high-risk group, 63% received statins, but 53% were not received on the recommended high-intensity statin.

**Conclusion:**

Our study revealed that 44% of eligible patients did not receive statins for primary ASCVD prevention, and 27% of those on statins had inappropriate intensity. With rising CVD-related deaths in PLWH, primary prevention of ASCVD is crucial. This project highlights the need for enhanced physician education on HIV-specific risks to improve statin use to reduce preventable CVD-related deaths.

**Disclosures:**

All Authors: No reported disclosures

